# Mitochondrial Replication Assay (MIRA) for Efficient in situ Quantification of Nascent mtDNA and Protein Interactions with Nascent mtDNA (mitoSIRF)

**DOI:** 10.21769/BioProtoc.4680

**Published:** 2023-05-20

**Authors:** Macy Lozen, Yue Chen, Rebecca A. Boisvert, Katharina Schlacher

**Affiliations:** Department of Cancer Biology, University of Texas MD Anderson Cancer Center, Houston, TX, USA

**Keywords:** MIRA, Mitochondria, Mitochondrial DNA, mtDNA replication, mtDNA instability, Proximity ligation assay, BRCA2, Fanconi anemia

## Abstract

Mitochondria play decisive roles in bioenergetics and intracellular communication. These organelles contain a circular mitochondrial DNA (mtDNA) genome that is duplicated within one to two hours by a mitochondrial replisome, independently from the nuclear replisome. mtDNA stability is regulated in part at the level of mtDNA replication. Consequently, mutations in mitochondrial replisome components result in mtDNA instability and are associated with diverse disease phenotypes, including premature aging, aberrant cellular energetics, and developmental defects. The mechanisms ensuring mtDNA replication stability are not completely understood. Thus, there remains a need to develop tools to specifically and quantifiably examine mtDNA replication. To date, methods for labeling mtDNA have relied on prolonged exposures of 5′-bromo-2′-deoxyuridine (BrdU) or 5′-ethynyl-2′-deoxyuridine (EdU). However, labeling with these nucleoside analogs for a sufficiently short time in order to monitor nascent mtDNA replication, such as under two hours, does not produce signals suited for efficient or accurate quantitative analysis. The assay system described here, termed Mitochondrial Replication Assay (MIRA), utilizes proximity ligation assay (PLA) combined with EdU-coupled Click-IT chemistry to address this limitation, thereby enabling sensitive and quantitative analysis of nascent in situ mtDNA replication with single-cell resolution. This method can be further paired with conventional immunofluorescence (IF) for multi-parameter cell analysis. By enabling monitoring nascent mtDNA prior to the complete replication of the entire mtDNA genome, this new assay system allowed the discovery of a new mitochondrial stability pathway, mtDNA fork protection. Moreover, a modification in primary antibodies application allows the adaptation of our previously described in **s**itu protein **I**nteractions with nascent DNA **R**eplication **F**orks (SIRF) for the detection of proteins of interest to nascent mtDNA replication forks on a single molecule level (**mitoSIRF**).

Graphical overview

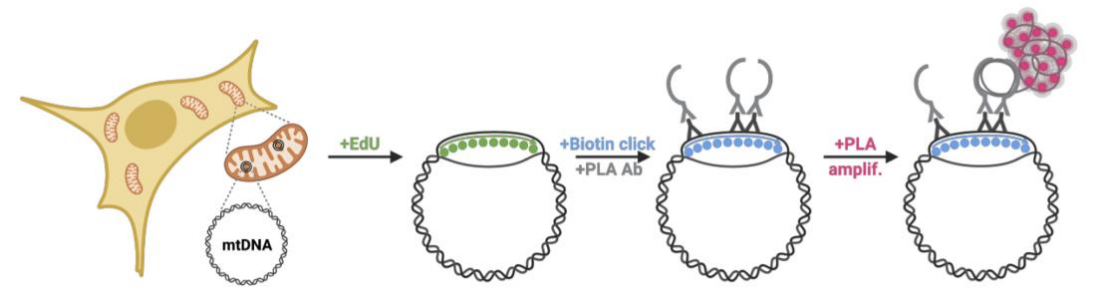

**Schematic overview of Mitochondrial Replication Assay (MIRA).** 5′-ethynyl-2′-deoxyuridine (EdU; green) incorporated in DNA is tagged with biotin (blue) using Click-IT chemistry. Subsequent proximity ligation assay (PLA, pink circles) using antibodies against biotin allows the fluorescent tagging of the nascent EdU and amplification of the signal sufficient for visualization by standard immunofluorescence. PLA signals outside the nucleus denote mitochondrial DNA (mtDNA) signals. Ab, antibody. In in situ protein interactions with nascent DNA replication forks (mitoSIRF), one of the primary antibodies is directed against a protein of interest, while the other detects nascent biotinylated EdU, thus enabling in situ protein interactions with nascent mtDNA.

## Background

Mitochondria, the organelles best known for their orchestration of cellular bioenergetics, operate semi-autonomously within eukaryotic cells and are key signaling hubs for intracellular communication (Friedman and Nunnari, 2014). Each mitochondrion harbors multiple copies of its ~16 kb circular genome, which encodes essential subunits of the electron transport chain as well as a set of transfer and ribosomal RNAs (Falkenberg et al., 2007). Mitochondrial DNA (mtDNA) is replicated separately from nuclear DNA (nDNA): the mitochondrial genome is replicated by its organelle-specific replisome, comprised of DNA polymerase POLγ and other nuclear-encoded proteins (Holt and Reyes, 2012; Gustafsson et al., 2016; Bailey and Doherty, 2017). mtDNA replication can occur at any cell cycle stage. Because of its proximity to events during oxidative phosphorylation, mtDNA is constantly exposed to reactive oxygen species, rendering it highly susceptible to oxidative DNA damage (Kang and Hamasaki, 2002). Failure to repair damage of the mitochondrial genome additionally results in instability of mtDNA and mitochondrial dysfunction, which is associated with premature aging, tumorigenesis, and neurological disorders (Trifunovic et al., 2004; Chatterjee et al., 2006; Ishikawa et al., 2008; Park and Larsson, 2011). Apart from mtDNA repair pathways, mtDNA stability is also regulated at the level of mtDNA replication with the exonuclease activity of POLγ polymerase and *MGME1* (mitochondrial genome maintenance exonuclease 1) degrading damaged mtDNA and generating new mtDNA (Torregrosa-Munumer et al., 2019).

To date, detailed mechanisms of mtDNA replication linking to stability are incompletely understood. A comprehensive understanding of these mechanisms is imperative to allow the development of effective and specific agents targeting disease outcomes. For this purpose, efficient tools to specifically monitor and quantifiably measure nascent mtDNA replication are needed. mtDNA is replicated primarily by POLγ within one to two hours, yet methods for labeling mtDNA have historically relied on prolonged exposures (up to 24 h) of 5′-bromo-2′-deoxyuridine (BrdU) or 5′-ethynyl-2′-deoxyuridine (EdU) (Clayton, 1982; Korhonen et al., 2004). Incorporation of these nucleoside analogs for labeling times sufficiently short to monitor nascent mtDNA replication before completion of the entire mtDNA genome, e.g. less than two hours, does not produce signals suited for efficient quantitative analysis (Luzwick et al., 2021). The assay system described here, termed Mitochondrial replication assay (MIRA), utilizes proximity ligation assay (PLA) as a means of signal amplification of the mtDNA-incorporated nucleoside analogs to overcome this limitation. Specifically, mtDNA is labeled with the thymidine analog EdU, which is then biotinylated using Click-IT chemistry (Moses and Moorhouse, 2007). The PLA technology was developed by Soderberg et al. (2008) for single-molecule protein–protein interaction studies, utilizing antibodies with oligonucleotide conjugates that can be ligated into a circular DNA molecule when two antibodies are in close proximity to each other (<40 nm). Once ligated, the DNA circle can be amplified by rolling circle DNA polymerase. After annealing of fluorescent DNA probes that are complementary to the DNA circle, this procedure results in a highly amplified fluorescent signal, readily detectable by conventional immunofluorescence (IF) microscopy. In MIRA, PLA is adapted to signal-amplify the nascent mtDNA signals by utilizing complementary antibodies against biotin (detecting biotinylated EdU). As with conventional EdU and BrdU labeling, MIRA also detects and visualizes nDNA. Nonetheless, only cytoplasmic MIRA signals are considered for analysis, which are present irrespective of the cell cycle state and do not form without EdU or in mtDNA-depleted Rho zero cells (Luzwick et al., 2021). With amplification, the nascent mtDNA can be readily detected and quantified with a 1 h EdU pulse, which is less time than needed to replicate the entire genome. This assays system enables the monitorization of ongoing mtDNA replication prior to completion of mtDNA genome replication. It therefore differs from most common mtDNA label schemes, which use prolonged nucleoside analog incorporation for extended time over multiple hours. Since the mtDNA is replicated within one to two hours, prolonged labeling will predominantly result in post-replicative signals. Moreover, as an adaptation from our single-cell assay for in **s**itu protein **I**nteractions with nascent DNA **R**eplication **F**orks (SIRF) protocol (Roy et al., 2018; Roy and Schlacher, 2019), MIRA can be combined with a primary antibody against a protein of interest to detect protein mtDNA interactions (mitoSIRF). The MIRA and mitoSIRF assay systems allowed the discovery of a new mitochondrial stability pathway, mtDNA fork protection (Luzwick et al., 2021). Specifically, BRCA and FANC tumor-suppressor proteins protect nascent mtDNA from instability during ongoing mtDNA replication; this would not have been detectable by previous assay systems that visualize post-replicative events. The instability is caused by MRE11-dependent nuclease degradation, which results in cGAS-mediated inflammatory signaling (Luzwick et al., 2021).

The MIRA method requires a minimal number of cells (~100–1,000), preserves the single-cell resolution as seen with IF, which provides valuable information regarding cell morphology, and can be readily and accurately quantified. The MIRA assay can furthermore be combined with conventional IF for multi-parameter biomarker analysis, including cell cycle stage or cell identity. If combined with other biomarkers, primary and secondary antibody staining for conventional IF is performed following the Click-IT and PLA reaction to avoid interference of PLA secondary antibodies from cross-reacting with IF primary antibodies.

## Materials and Reagents

1. 8-well chamber microscope slides (Thermo Scientific, Nunc, catalog number: 177402)
2. Plastic coverslips (Electron Microscopy Sciences, catalog number: 72261-50)
3. Glass coverslips (Fisher Scientific, catalog number: 12-548-5M)
4. Kimwipes (Fisher Scientific, catalog number: NC9855580)
5. Aluminum foil (Fisher Scientific, catalog number: 01-213-105)
6. Paper towel (Envision, catalog number: 23304)
7. 0.22 μm bottle top filter (Corning, catalog number: 430758)
8. Rabbit anti-biotin antibody (Cell Signaling, catalog number: 5597S)
9. Mouse anti-biotin antibody (Sigma-Aldrich, catalog number: B7653)
10. 5′-ethylene-2′-deoxyuridine (EdU) (Invitrogen, catalog number: A10044)
11. Paraformaldehyde 32% solution, EM grade (PFA) (EMS, catalog number: 15714)
12. Triton X-100 (Sigma-Aldrich, catalog number: T8787)
13. Biotin azide (Invitrogen, catalog number: B10184)
14. Alexa Fluor 488 azide (Invitrogen, catalog number: A10266)
15. Copper sulfate solution (Fluka Analytical, catalog number: 35185)
16. Sodium ascorbate (Sigma-Aldrich, catalog number: 11140)
17. Phosphate buffered saline (PBS) (Sigma-Aldrich, catalog number: P4417)
18. Duolink^®^ mouse plus PLA probe (Sigma-Aldrich, catalog number: DUO92001-100RXN)
19. Duolink^®^ rabbit minus PLA probe (Sigma-Aldrich, catalog number: DUO92005-100RXN)
20. Duolink^®^ blocking solution (Sigma-Aldrich, catalog number: DUO82007-8ML)
21. Duolink^®^ antibody diluent (Sigma-Aldrich, catalog number: DUO82008-8ML)
22. Duolink^®^ PLA detection reagent red (Sigma-Aldrich, catalog number: DUO92008-100RXN)
23. Duolink^®^ in situ wash buffers (Sigma-Aldrich, catalog number: DUO82049-20L)
24. 4’,6-diamidino-2-phenylindole (DAPI) (Life Technologies, catalog number: 62248)
25. Prolong Gold antifade reagent (Invitrogen, catalog number: P36934)
26. Cell-specific culturing media
27. Dideoxycytidine (ddC, 20 μM) (Sigma-Aldrich, catalog number: D5782)
28. MitoPQ (10 μM) (Abcam, catalog number: 1821370-28-8)
29. mtOX (Wisnovsky et al., 2016)
30. Sodium chloride (NaCl) (Fisher Scientific, catalog number: 7647-14-5)
31. Tween 20 (Sigma-Aldrich, catalog number: P7949)
32. Hydrochloric acid (HCl) (Fisher Scientific, catalog number: 7647-01-0)
33. Trizma hydrochloride (Tris-HCl) (Sigma-Aldrich, catalog number: T5941)
34. Tris base (Fisher Scientific, catalog number: BP152-1)
35. Dimethyl sulfoxide (DMSO) (Santa Cruz Biotechnology, catalog number: sc-358801)
36. Fixation solution (see Recipes)
37. Permeabilization solution (see Recipes)
38. Wash buffer A (see Recipes)
39. Wash buffer B (see Recipes)
40. EdU stock solution (see Recipes)
41. Biotin-azide stock solution (see Recipes)
42. Alexa488-azide stock solution (see Recipes)

## Equipment

1. Autoflow IR water jacketed CO_2_ incubator (NUAIRE, model: NU-4750)
2. Nikon eclipse Ti-U inverted microscope (Nikon, model: Ti-U)
3. Colin jar (Thermo Scientific^TM^ E94)
4. Fine curved forceps (Fine Science Tools, Dumont #7, catalog number: 11271-30)
5. Slide box (VWR, catalog number: 82003-414)
6. Vortexer (VWR analog vortex mixer, catalog number: 10153-838)
7. 4 °C refrigerator (BSI, model: SCGP21OW1AREF)
-20 °C freezer (BSI, model: ABT-2020MB)

## Software

1. NIS-elements (Nikon, https://www.nikoninstruments.com/Products/Software)
2. ImageJ (ImageJ, https://imagej.net/Welcome)
3. Microsoft Excel (Microsoft, https://products.office.com/en-us/excel)
4. GraphPad Prism (GraphPad, https://www.graphpad.com/scientific-software/prism/)

## Procedure

- **Cell labeling**
  1. One day before the experiment, plate 1 × 10^4^–2 × 10^4 ^cells growing in log-phase in 300 µL of appropriate growth medium in each well of an 8-well chamber microscope slide. Cells should reach 50%–60% confluency the day of the experiment.
*Note: We have only used this procedure with adherent cells. However, it should be possible to use suspension cells, as they can be treated with EdU in solution, deposited on microscope slides using a cytospin, and then fixed to proceed with the assay.*
  2. The next day, aspirate growth medium from chamber wells and add 200 µL of prewarmed growth medium containing 20 μM EdU. Incubate the slide for 1 h at 37 °C in a tissue culture incubator. Note that the length of incubation may need to be optimized for the cell line/type. This condition, *E only*, allows for detection of unperturbed newly replicated mtDNA.
*Note: Prewarm growth medium to 37 °C prior to treating cells, to avoid replication disruption of cells during treatments. For the same reason, be succinct with washes and treatments to limit exposure to room temperature (RT), which may disrupt replication.*
  3. (Optional) For assaying mtDNA fork protection, aspirate the media containing EdU after 1 h, wash chamber wells swiftly but gently with PBS (pH 7.4) at RT, aspirate, and add growth medium containing a mtDNA replication stalling agent of choice for a respective amount of time.
*Note: Typical mtDNA-specific replication stalling agents are ddC (20 μM), MitoPQ (10 μM), or mtOX (4 μM) added for 1–3 h at 37 °C before fixation. For assaying replication restart, which is the capacity of resuming mtDNA replication after replication stalling, treat with mtDNA stalling agent first, followed by swift PBS washes and incubation of EdU media.*
  4. Always include a well with no EdU treatments as a negative control—*No EdU*.
- **Cell fixation**
  1. Aspirate growth medium and gently wash wells twice with PBS at RT. Carefully add 200 μL of freshly prepared fixation solution (2% PFA diluted in PBS) to each well. Incubate at RT for 15 min without disturbing the slide.
*Note: Fixation time may vary with cell type. Handle PFA with caution inside a chemical safety cabinet. Discard PFA waste according to institutional biosafety guidelines. Other fixation methods may be possible but need to be tested.*
  2. After fixation, discard PFA and wash wells with PBS twice for 5 min each at RT. Disassemble chambers from the slide and remove silicone gasket using fine curved forceps (Figure 1). Ensure that the wells do not dry out by carefully placing them in a Coplin jar containing PBS as needed.
*Note: Fixed slides can be stored in PBS at 4 °C for up to one week.*
    **Figure 1.**
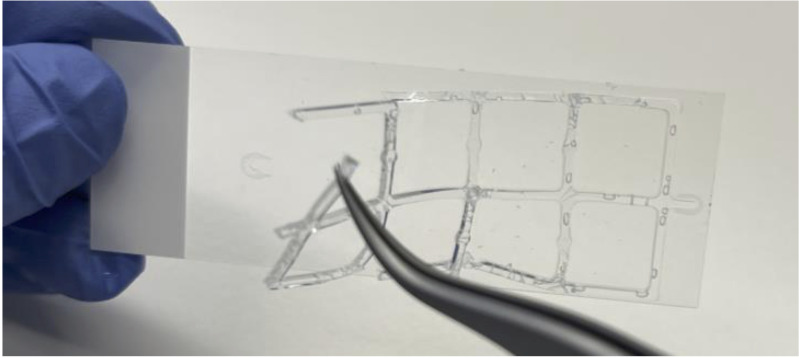
Silicone gasket removal. Removal of silicone gasket using fine curved forceps. Take caution to avoid scratching slide with the forceps.
- **Cell permeabilization**
  1. Permeabilize cells by placing slides in a Coplin jar containing 60 mL of permeabilization solution (0.25% Triton X-100 in PBS), enough to completely cover the slides for 15 min at RT.
    Next, wash slides three times in a Coplin jar containing PBS for 5 min at RT.
- **Click-IT reaction**
  1. Prepare a humidified chamber by placing wet paper towels or Kimwipes inside a slide box at RT (Figure 2). Let the chamber equilibrate for 5 min while closed.
    **Figure 2.**
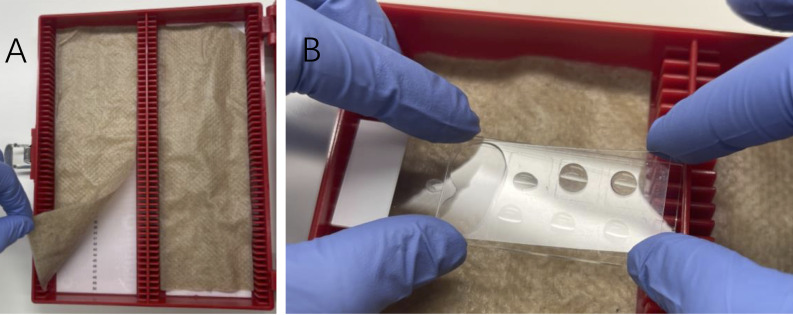
Humidified slide chamber. A. Preparation of a humidified chamber by placing folded paper towels or Kimwipes wetted with distilled water or PBS in an empty slide box. B. Slides are laid flat, facing up during incubations, and are carefully covered with plastic coverslips. Air bubbles can be avoided by applying the coverslip one end at a time.
  2. Prepare fresh Click-IT reaction cocktail. In the following order, add 2 mM copper sulfate to PBS, 10 μM biotin azide, and 100 mM sodium ascorbate. Mix well by vortexing after adding each component.
*Notes:*
    - *Make 1 M sodium ascorbate fresh before each use.*
    - *Protect biotin azide from light while handling.*
    - *A mixture of Alexa 488 azide and biotin azide (1:10, total 10 μM) can be used rather than biotin azide alone, to enable determination of cell-cycle phase (Figure 3). Protect Alexa 488 azide from light while handling.*
    **Figure 3.**
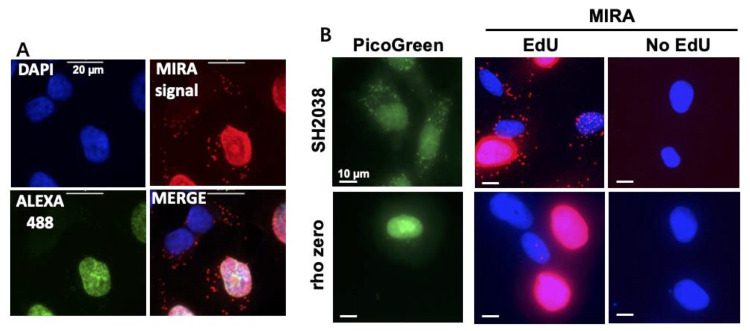
Representative image to distinguish MIRA signals at various stages of the cell cycle and controls. A. Example of MIRA in UWB1.289 cells treated with 20 μM EdU for 1 h. Note that the green nuclear signal for Alexa 488 azide co-click enables distinction of S-phase from non-S-phase cells [no green nuclear signal, DAPI signal (blue) only]. PLA signal (red) is detected inside the nucleus during S-phase and outside the nucleus in mitochondria during all cell cycle phases. Scale bars = 20 μm. B. Representative images of MIRA assay (red) in Rho zero SH2038 cells that are depleted of mtDNA as seen by PicoGreen DNA stain (green), with and without EdU as negative control. Scale bars = 10 μm.
  3. Place slides in the humidified chamber facing up (Figure 2). Add 40–50 μL/well of Click-IT reaction cocktail. Carefully cover each slide with a clean plastic coverslip, avoiding the formation of air bubbles.
*Note: Do not allow drying of the wells at any point during the protocol.*
  4. Close the chamber lid to maintain humidified conditions in the chamber and incubate at RT for 1 h.
- **Blocking and primary antibody incubation**
  1. Gently remove plastic coverslips and wash slides in a Coplin jar containing PBS three times for 5 min at RT.
  2. Place slides back in the humidified chamber. Add 50 μL/well of Duolink blocking solution. Carefully cover each slide with a clean plastic coverslip, avoiding air bubbles.
  3. Close the humidified chamber and incubate for 1 h at RT.
  4. Dilute primary antibodies [mouse anti-biotin (1:100) and rabbit anti-biotin (1:100)] in Duolink antibody diluent.
*Note: For mitoSIRF, replace either rabbit anti-biotin or mouse anti-biotin with a primary antibody against a protein of interest. Primary antibody concentrations and length of incubation can be adjusted for optimization.*
  5. After 1 h, gently remove plastic coverslips and tap slides to remove blocking solution.
  6. Place slides in the humidified chamber and directly add 35 μL/well of the diluted primary antibody solution. Apply new plastic coverslips, avoiding formation of air bubbles.
  7. Close humidified chamber and incubate at 4 °C overnight.
- **Proximity ligation assay (PLA)**
  1. Wash slides in a Coplin jar containing 60 mL of Duolink wash buffer A (10 mM Tris-HCl pH 7.5, 150 mM NaCl, and 0.05% Tween 20) three times for 5 min each at RT to remove primary antibody.
  2. Prewarm the humidified chamber by placing it at a 37 °C incubator during washes.
  3. Using the Duolink PLA detection kit, prepare Duolink in situ PLA probes (anti-mouse plus and anti-rabbit minus) by diluting them 1:5 in antibody diluent.
  4. Tap off excess Duolink wash buffer A and place slides in the prewarmed humidified chamber. Add 30 μL/well of PLA probes.
  5. Apply a new plastic coverslip to each slide and incubate at 37 °C for 1 h in the humidified chamber.
  6. Next, wash slides three times for 5 min each at RT in a Coplin jar containing Duolink wash buffer A to remove PLA probes.
  7. Using the Duolink PLA detection kit, prepare DNA ligation mix by diluting the 5× Duolink ligation buffer (1:5) and Duolink 1× ligase (1:40) in autoclaved water.
*Note: Vortex the ligation stock buffer during and after thawing, making sure to dissolve any precipitate. Keep ligase in a freezing block at -20 °C and add to ligation buffer.*
  8. Tap off excess wash buffer and place slides back in the humidified chamber. Add 30 μL/well of ligation mix.
  9. Put a new plastic coverslip on each slide and incubate in the humidified chamber at 37 °C for 30 min.
  10. Wash slides in a Coplin jar with Duolink wash buffer A two times for 5 min each at RT.
  11. Prepare amplification mix by diluting the 5× Duolink amplification buffer (1:5) and Duolink 1× polymerase (1:80) in autoclaved water.
*Note: Limit the exposure of the amplification buffer to light, e.g., by preparing the solution in an amber light-protected tube, or in a tube covered with aluminum foil. Keep polymerase in a freezing block at -20 °C.*
  12. Tap off excess wash buffer and place slides back in the humidified chamber. Add 30 μL/well of amplification mix.
  13. Cover each slide with a new plastic coverslip and incubate in the humidified chamber at 37 °C for exactly 100 min.
  14. Wash slides in a Coplin jar containing Duolink wash buffer B (200 mM Tris-HCl pH 7.5 and 100 mM NaCl) three times for 10 min each at RT and in the dark.
  15. Wash slides in a Coplin jar containing 0.01× diluted Duolink wash buffer B for 1 min in the dark to remove excess salt.
- **DAPI staining and mounting**
  1. Prepare DAPI solution (1:1,000, 1 μg/mL end concentration) in PBS.
*Note: Protect from light.*
  2. Tap off excess wash buffer B and place slides back in the humidified chamber. Add 30 μL/well of DAPI solution.
  3. Cover with new plastic coverslips and incubate in the humidified chamber at RT for 5 min.
  4. Wash slides in a Coplin jar containing PBS two times for 5 min each at RT.
  5. Tap off excess PBS from slides and place them on a paper towel for mounting.
  6. Add ~20 μL of Prolong Gold antifade reagent to each well of the slide and mount with glass coverslips (1.5 mm). Avoid air bubbles while mounting and wipe off excess mounting reagent from the edges of the coverslip. Keep slides in a dry slide box at RT overnight to cure and avoid exposure to light.
*Note: Should be imaged within two days. If not feasible, store slides at -20 °C, allow slides to come to RT before imaging, and do not freeze slides repeatedly.*

## Data analysis

1. Image slides using a fluorescent microscope (such as Nikon Eclipse Ti) at a magnification of 40×/0.95 numerical aperture (N.A.) (20×/0.75 N.A. may also be sufficient for larger cells). PLA signals are captured in the TXRED channel (Ex 594 nm, Em 624 nm, as recommended by Sigma Duolink for Duolink detection reagent red). The GFP filter is used to image cells when co-clicked with Alexa 488 azide (Ex 495 nm, Em 519 nm). DAPI filter is used to visualize cell nuclei (Ex 358 nm, Em 461 nm). The merged image file is used for subsequent analysis (Figure 3). Typically, 4–8 image fields in different areas of the well are obtained for a total of 50–150 cells per condition. The microscope intrinsic quantitation software, such as Nikon NIS-elements software (bright spot detection) or ImageJ (https://imagej.net/Welcome), can be used to determine the number of MIRA PLA or mitoSIRF signals per cell. Hereby, only signals outside the nucleus are considered. Alternatively, when signals are very abundant and indistinguishable by the software, the mean fluorescent intensity of the TXRED channel is measured using the microscope software, such as Nikon NIS-elements software.
2. Counting signals outside of the nucleus of cells containing Alexa 488 allows distinction between mitochondrial events in S-phase from those in non-S-phase.
3. A student t-test is used to determine the significance of the difference between conditions and/or cell lines. If the data follows a non-normal distribution as measured by the D'Agostino & Pearson normality test, a Mann-Whitney t-test is used to determine the statistical significance (Luzwick et al., 2021).

## Recipes

1. **Fixation solution**
  - Add 10 mL of 32% PFA solution stock in 150 mL of PBS pH 7.4 to make 2% PFA and mix well.
  - Store leftover diluted PFA in air-tight bottles, protected from light at either RT (for up to one week) or 4 °C for up to one month.
2. **Permeabilization solution**
  - Add 12.5 mL of 10% Triton X-100 solution in 487.5 mL of PBS (pH 7.4) to make 0.25% Triton X-100 solution.
  - Mix well and store at RT.
3. **Wash buffer A**
  - Dissolve 8.8 g of NaCl, 1.2 g of Tris base, and 0.5 mL of Tween 20 in 800 mL of autoclaved water.
  - Adjust pH to 7.4 using HCl.
  - Add autoclaved water to 1,000 mL (final concentrations 0.01 M Tris, 0.15 M NaCl, and 0.05% Tween 20).
  - Filter the solution (0.22 μm filter) and store at 4 °C. Bring the solutions to RT before use.
4. **Wash buffer B**
  - Dissolve 5.84 g of NaCl, 4.24 g of Tris base, and 26 g of Tris-HCl in 500 mL of autoclaved water.
  - Adjust pH to 7.5 using HCl.
  - Add autoclaved water to 1,000 mL (final concentrations 0.2 M Tris and 0.1 M NaCl).
  - Filter the solution (0.22 μm filter) and store at 4 °C. Bring the solutions to RT before use.
5. **EdU stock solution**
  Add 2 mL of DMSO directly into the vial containing 50 mg of EdU to make 100 mM stocks. Dissolve powder by vortexing. Store aliquots of stock solution at -20 °C protected from light.
6. **Biotin-azide stock solution**
  Add 1.623 mL of DMSO directly into the vial containing 1 mg of biotin azide to make 1 mM stocks. Dissolve powder by vortexing. Store aliquots of stock solution at -20 °C, desiccate, and protect from light.
7. **Alexa488-azide stock solution**
  Add 0.58 mL of DMSO directly into the vial containing 0.5 mg of Alexa 488 azide to make 1 mM stocks. Dissolve powder by vortexing. Store aliquots of stock solution at -20 °C, desiccate, and protect from light.

## References

[1] Bailey L. J. and Doherty A. J.(2017). Mitochondrial DNA replication: a PrimPol perspective. Biochem Soc Trans 45(2): 513-529. 28408491 10.1042/BST20160162 PMC5390496

[2] Chatterjee A., Mambo E. and Sidransky D.(2006). Mitochondrial DNA mutations in human cancer. Oncogene 25(34): 4663-4674. 16892080 10.1038/sj.onc.1209604

[3] Clayton D. A.(1982). Replication of animal mitochondrial DNA. Cell 28(4): 693-705. 6178513 10.1016/0092-8674(82)90049-6

[4] Falkenberg M., Larsson N. G. and Gustafsson C. M.(2007). DNA replication and transcription in mammalian mitochondria. Annu Rev Biochem 76: 679-699. 17408359 10.1146/annurev.biochem.76.060305.152028

[5] Friedman J. R. and Nunnari J.(2014). Mitochondrial form and function. Nature 505(7483): 335-343. 24429632 10.1038/nature12985 PMC4075653

[6] Gustafsson C. M., Falkenberg M. and Larsson N. G.(2016). Maintenance and Expression of Mammalian Mitochondrial DNA. Annu Rev Biochem 85: 133-160. 27023847 10.1146/annurev-biochem-060815-014402

[7] Holt I. J. and Reyes A.(2012). Human mitochondrial DNA replication. Cold Spring Harb Perspect Biol 4(12). 10.1101/cshperspect.a012971 PMC3504440 23143808

[8] Ishikawa K., Takenaga K., Akimoto M., Koshikawa N., Yamaguchi A., Imanishi H., Nakada K., Honma Y. and Hayashi J.(2008). ROS-generating mitochondrial DNA mutations can regulate tumor cell metastasis. Science 320(5876): 661-664. 18388260 10.1126/science.1156906

[9] Kang D. and Hamasaki N.(2002). Maintenance of mitochondrial DNA integrity: repair and degradation. Curr Genet 41(5): 311-322. 12185497 10.1007/s00294-002-0312-0

[10] Korhonen J. A., Pham X. H., Pellegrini M. and Falkenberg M.(2004). Reconstitution of a minimal mtDNA replisome in vitro. EMBO J 23(12): 2423-2429. 15167897 10.1038/sj.emboj.7600257 PMC423294

[11] Luzwick J. W., Dombi E., Boisvert R. A., Roy S., Park S., Kunnimalaiyaan S., Goffart S., Schindler D. and Schlacher K.(2021). MRE11-dependent instability in mitochondrial DNA fork protection activates a cGAS immune signaling pathway. Sci Adv 7(51): eabf9441. 34910513 10.1126/sciadv.abf9441 PMC8673762

[12] Moses J. E. and Moorhouse A. D.(2007). The growing applications of click chemistry. Chem Soc Rev 36(8): 1249-1262. 17619685 10.1039/b613014n

[13] Park C. B. and Larsson N. G.(2011). Mitochondrial DNA mutations in disease and aging. J Cell Biol 193(5): 809-818. 21606204 10.1083/jcb.201010024 PMC3105550

[14] Roy S., Luzwick J. W. and Schlacher K.(2018). SIRF: Quantitative in situ analysis of protein interactions at DNA replication forks. J Cell Biol 217(4): 1521-1536. 29475976 10.1083/jcb.201709121 PMC5881507

[15] Roy S. and Schlacher K.(2019). SIRF: A Single-cell Assay for in situ Protein Interaction with Nascent DNA Replication Forks. Bio Protoc 9(18): e3377. 10.21769/BioProtoc.3377 PMC7854004 33654873

[16] Soderberg O., Leuchowius K. J., Gullberg M., Jarvius M., Weibrecht I., Larsson L. G. and Landegren U.(2008). Characterizing proteins and their interactions in cells and tissues using the in situ proximity ligation assay. Methods 45(3): 227-232. 18620061 10.1016/j.ymeth.2008.06.014

[17] Torregrosa-Munumer R., Hangas A., Goffart S., Blei D., Zsurka G., Griffith J., Kunz W. S. and Pohjoismaki J. L. O.(2019). Replication fork rescue in mammalian mitochondria. Sci Rep 9(1): 8785. 31217442 10.1038/s41598-019-45244-6 PMC6584726

[18] Trifunovic A., Wredenberg A., Falkenberg M., Spelbrink J. N., Rovio A. T., Bruder C. E., Bohlooly Y. M., Gidlof S., Oldfors A., Wibom R., .(2004). Premature ageing in mice expressing defective mitochondrial DNA polymerase. Nature 429(6990): 417-423. 15164064 10.1038/nature02517

